# Studies on the antihypertensive and antidyslipidemic activities of *Viola odorata *leaves extract

**DOI:** 10.1186/1476-511X-11-6

**Published:** 2012-01-10

**Authors:** Hasan S Siddiqi, Malik H Mehmood, Najeeb U Rehman, Anwar H Gilani

**Affiliations:** 1 Natural Product Research Division, Department of Biological and Biomedical Sciences, The Aga Khan University Medical College, Karachi-74800, Pakistan; 2 Department of Pharmacology, Faculty of Pharmacy, University of Karachi, Karachi, Pakistan

**Keywords:** *Viola odorata *leaves, antihypertensive, antidyslipidemic, Ca^++ ^antagonist, NO-mediated.

## Abstract

**Background:**

This study was undertaken to provide pharmacological basis for the medicinal use of *Viola odorata *Linn. in hypertension and dyslipidemia using the *in vivo *and *in vitro *assays.

**Results:**

*Viola odorata *leaves extract (Vo.Cr), which tested positive for alkaloids, saponins, tannins, phenolics, coumarins and flavonoids, caused a dose-dependent (0.1-1.0 mg/kg) decrease in mean arterial blood pressure in anaesthetized rats. In isolated guinea-pig atria, Vo.Cr equally inhibited force and rate of spontaneous atrial contractions. On the baseline of rat thoracic aortae (endothelium-intact and denuded), the plant extract caused phentolamine-sensitive vasoconstriction. When tested on phenylephrine (PE, 1 μM) and K^+ ^(80 mM)-induced vasoconstriction, Vo.Cr caused a concentration-dependent relaxation and also caused a rightward shift of Ca^++ ^concentration-response curves as well as suppression of PE (1 μM) control peaks in Ca^++^-free medium, similar to that caused by verapamil. In the presence of L-NAME, the relaxation curve of Vo.Cr was partially inhibited showing involvement of Nitric oxide (NO) mediated pathway. In Tyloxapol-induced dyslipidemia, Vo.Cr caused reduction in total cholesterol and triglyceride levels. In high-fat diet-induced dyslipidemia model, the plant extract caused a significant decrease in total cholesterol, LDL-C, atherogenic index and prevented the increase in average body weights, while it increased HDL-C.

**Conclusions:**

These data indicate that the vasodilator effect of the plant extract is mediated through multiple pathways like inhibition of Ca^++ ^influx via membranous Ca^++ ^channels, its release from intracellular stores and NO-mediated pathways, which possibly explain the fall in BP. The plant also showed reduction in body weight and antidyslipidemic effect which may be due to the inhibition of synthesis and absorption of lipids and antioxidant activities. Thus, this study provides a pharmacologic rationale to the medicinal use of *Viola odorata *in hypertension and dyslipidemia.

## Background

Hypertension and dyslipidemia, major risk factors for coronary heart disease [1], are interrelated. The overall dyslipidemia can contribute to a chronic increase in vascular tone resulting in hypertension [2]. In order to reduce cardiovascular risk, it is important to regulate hypertension as well as dyslipidemia [3].

The modern pharmacological therapy is costly and associated with multiple side effects resulting in patient non-compliance. Thus there is a need to explore alternative therapies particularly from herbal sources as these are cost effective and possess minimal side-effects.

*Viola odorata *Linn., commonly known as sweet violet in English, belongs to the family Violaceae. It is called Banafsha in Indo-Pakistan [4]. The plant is native to Asia, North Africa and Europe. In Pakistan, *Viola odorata *is found in the northern areas, such as Nathia gali, Hazara, Kaghan, Swat and Chitral at the height of 1500-2000 meters [5]. Its history as a medicinal herb dates back as far as 500 BC, where it was known to be used to relieve pain due to cancer [6]. In the traditional system, it has been used in anxiety [7], insomnia and to lower blood pressure [8].

*Viola odorata *contains alkaloid, glycoside, saponins, methyl slicylate, mucilage and vitamin C [9]. The plant has been reported to possess antioxidant [10] and diuretic [11] activities along with other beneficial effects but no study has been found regarding its blood pressure lowering or lipid-lowering activity. In present investigation, we report the antihypertensive and antidyslipidemic effects of *Viola odorata *along with possible mechanisms.

## Materials and methods

### Plant material and Extraction procedure

Dried leaves of *Viola odorata *were purchased from "Insaf kiryana store" Daryalal street, Jodia bazaar, Karachi, Pakistan. A sample of the plant material was deposited in the Natural Products Research Division, Department of Biological and Biomedical Sciences, The Aga Khan University, Karachi, Pakistan with voucher number VO-LF-06-10-107. The leaves, after cleaning of adulterants, weighed 1.3 kg, were soaked in 14 L of aqueous-methanol (30:70) for a total of 3 days with occasional shaking. The plant material was filtered through a muslin cloth and then through a Whatman qualitative grade-1 filter paper. This procedure was repeated twice and the combined filtrate was concentrated in a rotary evaporator at 35-40°C under reduced pressure to obtain a thick, dark brown extract (Vo.Cr) with a yield of 21% (w/w). Vo.Cr was completely solubilized both in distilled water and saline for use in the *in vitro *and *in vivo *experiments.

### Drugs and standards

Acetylcholine chloride, Nω-nitro-L-arginine methyl ester hydrochloride (L-NAME), cholesterol, cholic acid, phenylephrine, phentolamine hydrochloride, norepinephrine, verapamil hydrochloride, potassium chloride and tyloxapol of reagent grade were purchased from Sigma Aldrich Chemical Company (St Louis, MO, USA). Randox diagnostic kits for serum analyses were purchased from Randox Laboratories (Antrim, UK). The butter fat used was in the form of "Asli desi ghee" manufactured by Haleeb food industries, Pakistan purchased from a local bakery in Karachi, Pakistan. All chemicals used were of the highest purity grade. Physiological salt solutions were prepared fresh in distilled water on the day of each experiment, whereas stock solutions of all the drugs and extract were prepared in distilled water or saline and the dilutions were prepared fresh on the day of each experiment.

### Animals

Animals used in this study, Sprague-Dawley (SD) rats (180-200 g), Balb-C mice (20-25 g) and guinea-pigs (450-500 g) of either sex or local breed were housed at the Aga Khan University animal house under a controlled environment (23-25°C) with free access to food and water. All experiments performed complied with the rulings of the Institute of Laboratory Animal Resources, Commission on Life Sciences, National Research Council [12].

### Preparation of diets

The following two types of diet were prepared:

A. Normal diet: The normal diet was prepared as described previously by Harkness and Wagner [13] at the animal house of the Aga Khan University, Karachi. The standard diet consisted of flour (5 kg), chokar (5 kg), molasses (150 g), salt (75 g), nutrivet-L (33 g), potassium metabisulfite (15 g), oil (500 g), fishmeal (2.25 kg) and powdered milk (2 kg), comprising a total mass of about 15 kg of food material.

B. Atherogenic diet: Cholesterol (2% w/w), cholic acid (0.5% w/w) and butter fat (5% w/w) were added to the normal diet, as described by Ichihashi [14], with slight modifications.

All measures were taken to ensure the uniform mixing of additives in dry ingredients of the diet before kneading.

### Phytochemical screening

Preliminary screening of the plant extract for various phytochemical classes was carried out based on a modified version of the reported methods [15]. The crude extract was screened for the presence of saponins, flavonoids, tannins, phenols, coumarins, sterols, terpenes, alkaloids and anthraquinones.

### *In vivo *blood pressure measurement in anaesthetized rats

These experiments were performed on male Sprague-Dawley (SD) rats (200-250 g) as described previously [16]. The animals were anaesthetized with an intra-peritoneal (i.p.) injection of sodium thiopental (Pentothal, 70-90 mg/kg body weight), and arterial blood pressure was recorded through carotid artery cannulation by a pressure transducer (MLT1199) coupled to a Bridge Amplifier and PowerLab 4/25 (ADInstruments). Drugs were injected through a cannula inserted into the jugular vein. After a 20-min period of equilibration equilibrium, the rats were injected intravenously with 0.1 ml saline (0.9% NaCl) or with the same volume of test substance. Arterial pressure was allowed to return to the resting level between injections. Control responses of standards, such as acetylcholine (1 mg/kg) and norepinephrine (1 mg/kg), were obtained before the extracts were tested. Changes in mean arterial pressure (MAP) were recognized as the difference between the steady-state value before and the lowest reading after injection. MAP was calculated as the sum of the diastolic pressure (DP) and one-third of the pulse pressure (PP), where PP = SP-DP (SP = systolic pressure).

### Isolated tissue experiments

#### Guinea-pig atria

Guinea-pigs were sacrificed by cervical dislocation; right atria were dissected out carefully, cleaned of fatty tissue and mounted individually in 20 ml tissue baths containing Kreb's solution at 32°C and aerated with carbogen (5% CO_2 _in O_2_) [17]. The composition of the Kreb's solution was (mM): NaCl 118.2, NaHCO_3 _25.0, CaCl_2 _2.5, KCl 4.7, KH_2_PO_4 _1.3, MgSO_4 _1.2 and glucose 11.7 (pH 7.4). The tissues were allowed to beat spontaneously (due to the presence of pacemaker cells) under the resting tension of 1 g. An equilibrium period of 30 min was given before the application of any drug. Control responses of isoproterenol (1 μM) and of acetylcholine (1 μM) were obtained. Tension changes in the tissue were recorded via force-displacement transducer (model FT-03) using Grass Model 7 Polygraph.

#### Rat aorta preparation

The procedure of Furchgott and Zawadski [18] was followed with some modifications. Thoracic aorta was isolated from the rat and cut into rings, which were mounted individually in 5 ml of tissue bath, maintained at 37°C and aerated with carbogen. A preload of 1 g was applied to each preparation and incubated for 60 min. Changes in isometric tension were recorded and analyzed through a force transducer (model FORT100) coupled to a Trans-bridge (model TBM_4M_, World Precision Instruments, Hertfordshire, UK), a PowerLab data acquisition system (model ML845, ADInstruments) and a computer running the Chart software (version 5.3). The tissues were then stabilized with phenylepherine (PE 1 μM). After stabilization, an induced contraction was obtained with PE. Once plateau was achieved, acetylcholine (ACh 0.3 μM) was tested on PE-induced contraction to observe the endothelium integrity. The endothelium lining of some tissues was removed by gentle rubbing, which resulted in the disappearance of the relaxation caused by ACh. To study whether or not the vasodilator effect of the test substance is endothelium-dependent, the PE-induced contraction was pre-incubated with L-NAME (0.1 mM) for 20 min to explore the possible mode of endothelium-dependent vasodilator action [19].

High K^+ ^(80 mM) was also used to produce sustained contraction. The inhibition of PE and high K^+ ^-induced sustained contractions would indicate a blockade of Ca^++ ^influx through membrane bound receptor-operated and voltage-dependent Ca^++ ^channels respectively [20]. The plant material was then tested against PE-evoked peaks in the Ca^++^-free Kreb's solution to observe its effect on the intracellular stores. In the Ca^++^-free medium, PE acts through stimulation of α_1_-adrenergic receptors and then the consequent conversion of phosphatidylinositol to inositol-1, 4, 5-triphosphate which releases Ca^++ ^from the sarcoplasmic reticulum resulting in a tonic contraction [21].

To confirm the calcium channel blocking (CCB) activity, Ca^++ ^concentration-response curves (CRCs) were constructed in a Ca^++ ^free medium. Subsequently, the effect of increasing concentration of the extract was determined on the Ca^++ ^CRCs. A shift in the Ca^++ ^curves to the right would confirm CCB.

Some experiments were performed on the resting baseline of the endothelium intact as well as denuded preparations. The tissues were stabilized with PE. At a steady-state baseline, the vasoconstrictor effect of Vo.Cr was evaluated and expressed as percent of PE-induced contraction.

### Antidyslipidemic activities

#### Tyloxapol-induced dyslipidemia

The tyloxapol-induced dyslipidemic model [22] was followed with slight modifications. Male SD rats weighing 160-180 g were randomly divided into six groups (n = 6 each). All groups were fed normal diet. Group 3-6 were given treatment by oral gavage for ten days. Group 3 received atorvastatin 10 mg/kg; group 4 received Vo.Cr 150 mg/kg; group 5 received Vo.Cr 300 mg/kg and group 6 received Vo.Cr 600 mg/kg; all dissolved in drinking water. After 10 days of the treatment, all animals were fasted for 7 h and group 1 received saline (10 ml/kg; i.p.) whereas all other groups were administered tyloxapol (500 mg/kg; i.p.). On the following day, all animals were anaesthetized by diethyl ether (by inhalation), and the blood was collected for analysis of serum total cholesterol (TC) and triglycerides (TGs).

#### High-fat diet induced dyslipidemia

The effect of Vo.Cr on high fat diet-induced dyslipidemia was studied using the method described by Berroughui [23] with slight modifications introduced after pilot studies. Adult SD rats (12-14 weeks old, weighing 140-160 g) were randomly divided into five groups (7-10 in each). Group 1 was fed diet A (served as normal control) while all other groups were fed diet B (atherogenic diet). Group 2 served as dyslipidemic control. Groups 3-5 were given treatment by oral gavage for six weeks. Group 3 received atorvastatin 10 mg/kg; group 4 received Vo.Cr 300 mg/kg and group 5 received Vo.Cr 600 mg/kg; all dissolved in drinking water. Animals in all groups had free access to water and food. Diet consumption was monitored daily, and the gain in body weight was monitored weekly. At the end of 6 weeks of treatment, animals were fasted for 16 h before blood collection, and samples were analyzed for serum lipids and glucose levels.

### Biochemical studies

#### Estimation of lipid profile and glucose level

The blood was collected in vacuutainer by cardiac puncture from fasting anaesthetized rats. The serum was separated after centrifugation at 3000 rpm for 10 min. The serum lipids and glucose were assayed enzymatically using commercially available kits. Methods described by the manufacturer (Randox Laboratories Ltd., Co. Antrim, UK.) were used for the determination of serum total cholesterol (TC), high density lipoprotein (HDL), triglyceride (TG) and glucose. Low-density lipoproteins (LDL) were estimated indirectly by using formula: LDL = TC-HDL-TG/5. Atherogenic index was calculated using formula: Atherogenic index = TC-HDL/HDL [24].

#### Acute toxicity assessment

Vo.Cr was evaluated for acute toxicity using Balb-C mice as described earlier by Gilani [25]. The mice were divided into four groups of six mice in each group and were given increasing doses of Vo.Cr extract (1, 3 and 5 g/kg) in 10 ml/kg volume orally. A negative control group was administered saline (10 ml/kg). All the mice were allowed food and water *ad libitum *and were kept under regular observation for mortality and gross behavioural changes such as activity in home cage, apathy and aggression for 48 h.

### Statistical analysis

All data were expressed as means ± standard error of mean (SEM, n = number of experiments) and the median effective concentration (EC_50_) values were calculated as the geometric mean with 95% confidence intervals (CI). The statistical parameter applied was student's *t*-test. CRCs were analyzed by nonlinear regression. One-way analysis of variance (one-way ANOVA) followed by Tukey's post-test was used to determine significant differences in various biological parameters with and without treatment. Two-way ANOVA followed by Bonferroni's post-test correction was used for multiple comparisons of CRCs with control. *P*-values < 0.05 were considered significant. All the graphing, calculations and statistical analyses were performed using GraphPad Prism software version 4.00 for Windows, (GraphPad Software, San Diego, CA, USA, http://www.graphpad.com).

## Results

### Phytochemical screening

The phytochemical analysis of the crude extract of *Viola odorata *(Vo.Cr) showed the presence of alkaloids, saponins, tannins, phenolics, coumarins and flavonoids.

### Effect on blood pressure of anaesthetized rats

**Figure 1:**
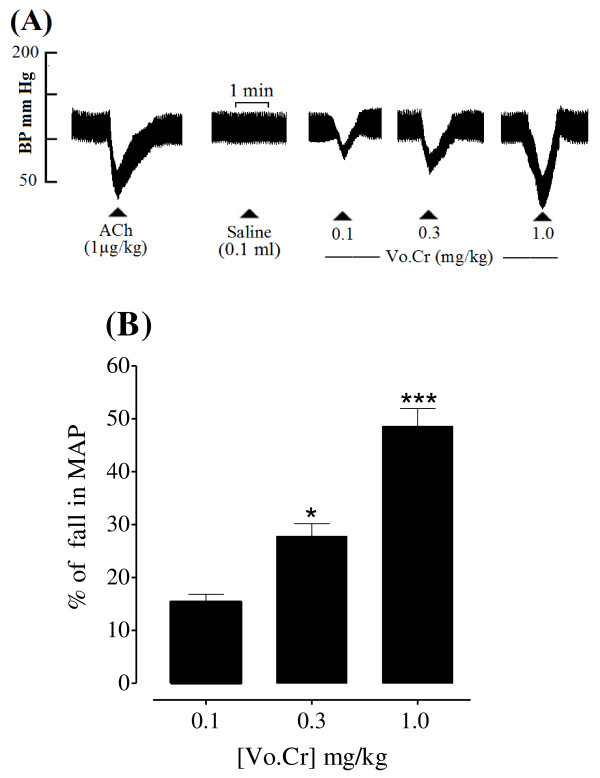
**(A) Tracing from a typical experiment showing the effect of increasing doses of crude extract of *Viola odorata *(Vo.Cr) on mean arterial blood pressure (MAP) of an anaesthetized rat. **Small triangles indicate the times at which the drugs were administered. (B) Bar chart represents combined effect (mean ± SEM) of 5 experiments. * *P *< 0.05; *** *P *< 0.001 (One way ANOVA followed by Tukey's multiple comparison).

Vo.Cr at 0.1, 0.3 and 1.0 mg/kg induced a percent fall of 15.40 ± 1.43, 27.80 ± 2.37 and 48.60 ± 3.35 (mean ± SEM, n = 5), respectively in mean arterial pressure (MAP) of rats under anaesthesia as shown in Figure 1.

### Effect on guinea-pig atria

**Figure 2:**
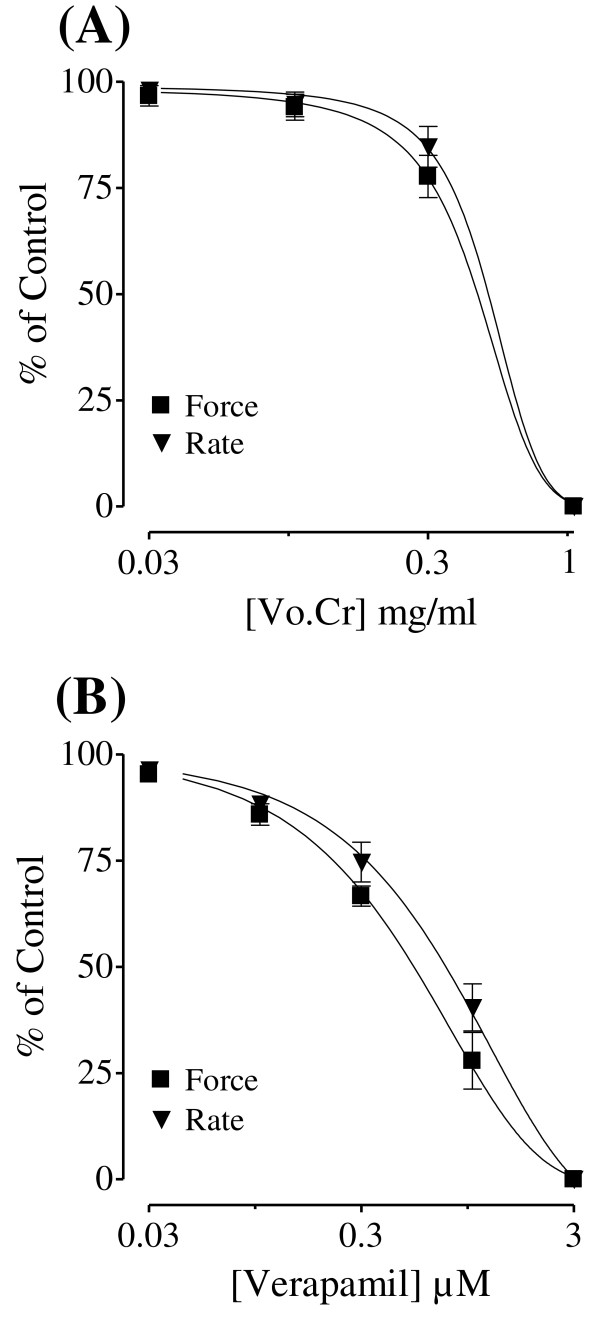
**The concentration-response curves showing inhibitory effect of (A) *Viola odorata *crude extract (Vo.Cr) and (B) verapamil on force and rate of contraction of isolated guinea-pig atrial preparations**. Values shown are mean ± SEM; n = 3-6.

In the isolated guinea-pig atrium, the plant extract depressed the force and rate of spontaneous contracting atria with respective EC_50 _values of 0.39 (0.21-0.75, 95% CI; n = 3) and 0.40 mg/ml (0.12-1.38; n = 3) as seen in Figure 2A, similar to verapamil which inhibited the force and rate of atrial contraction with respective EC_50 _values of 0.59 (0.47-0.75; n = 6) and 0.94 μM (0.68-1.30; n = 6) (Figure 2B).

### Effect on isolated rat aorta preparations

#### Effect on baseline tension

**Figure 3:**
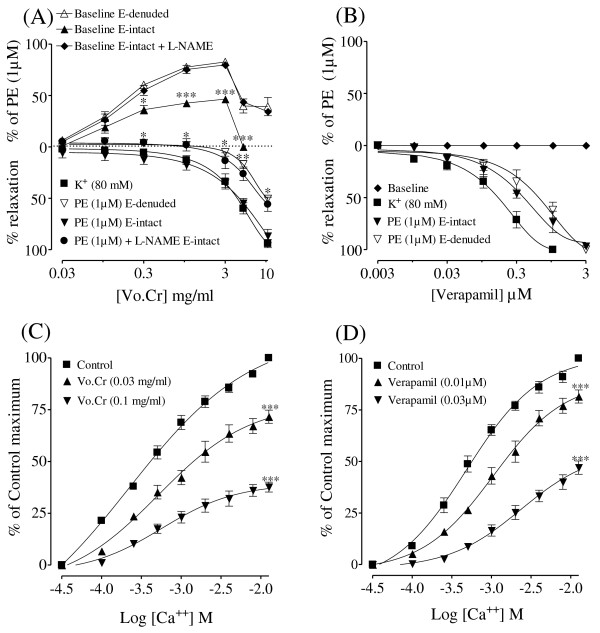
**(A) Effect of *Viola odorata *crude extract (Vo.Cr) and (B) verapamil on baseline tension and on high K^+ ^and phenlyephrine (PE)-induced contractions in the absence and presence of L-NAME in endothelium (E)- intact and E-denuded isolated rat aortic preparations**. The responses on baseline are expressed as a percentage of PE-induced contraction. (C) The concentration-response curves of Ca^++ ^in the absence and presence of increasing concentrations of Vo.Cr and (D) verapamil in isolated rat aortic preparations. Values shown are mean ± SEM; n = 4-7. In (A) and (B), *P < 0.05, ** *P *< 0.01 and *** *P *< 0.001 represent comparison of responses on E- intact preparations in the absence vs. presence of L-NAME (Student's *t*-test). In (C) and (D), *** *P *< 0.001 shows a comparison with control maximum (Two way ANOVA followed by Bonferroni's post-test correction).

When tested on resting tension of endothelium-intact tissue, Vo.Cr caused a concentration-dependent (0.03-3.0 mg/ml) vasoconstriction with maximum of 46.4 ± 2.8% (n = 4) of PE (1 μM) maximum contraction (Figure 3A) followed by complete relaxation at the next higher concentration of 5 mg/ml. In denuded tissues, Vo.Cr also induced vasoconstriction with resultant maximum contraction of 82.6 ± 2.6% (n = 4) at 3 mg/kg (Figure 3A) followed by partial relaxation at the dose of 5 mg/ml. When the vasoconstrictor effect of Vo.Cr in intact tissues was reproduced in the presence of L-NAME (0.1 mM), it was enhanced as 79.6 ± 2.9% (n = 4) vs 46.4 ± 2.8% (n = 4) (Figure 3A), while the vasodilator effect was partially inhibited at the higher tested concentration of 5 mg/ml. The vasoconstrictor effect of Vo.Cr, in intact and denuded tissues, was completely blocked when repeated in the presence of phentolamine (1 μM) (data not shown), while verapamil was devoid of any stimulatory effect on the baseline in either tissue (Figure 3B).

### Endothelium-dependent and independent effects

Vo.Cr caused a concentration-dependent relaxation of PE (1 μM)-induced contractions in endothelium-intact and denuded rat aortic preparations with respective EC_50 _values of 4.98 (3.93-6.30; n = 5) and 10.54 mg/ml (9.10-12.21; n = 4). When the relaxant effect of Vo.Cr was reproduced in intact aortic rings in the presence of L-NAME (0.1 mM), the relaxation curve was partially inhibited with resultant EC_50 _value of 10.11 mg/ml (6.61-15.43; n = 4) vs 4.98 (3.93-6.30; n = 5). When tested on high K^+ ^(80 mM)-induced contraction, Vo.Cr caused relaxation with EC_50 _value of 4.38 mg/ml (3.87-4.95; n = 7) as shown in Figure 3A. Verapamil also inhibited PE-induced contractions in endothelium-intact and denuded rat aortic preparations at similar concentrations with respective EC_50 _values of 0.41 (0.25-0.66; n = 3) and 0.71 μM (0.40-1.26; n = 3), while it inhibited high K^+^-induced contractions with EC_50 _value of 0.18 μM (0.13-0.26; n = 4) (Figure 3B).

### Determination of calcium channel blocking (CCB) activity

Pretreatment of rat aortic rings with Vo.Cr caused a concentration-dependent (0.03-0.1 mg/ml) rightward shift in the Ca^++ ^concentration-response curves constructed in Ca^++^- free medium, similar to that caused by verapamil at 0.01-0.03 μM (Figure 3).

### Effect on intracellular Ca^++ ^stores

**Figure 4:**
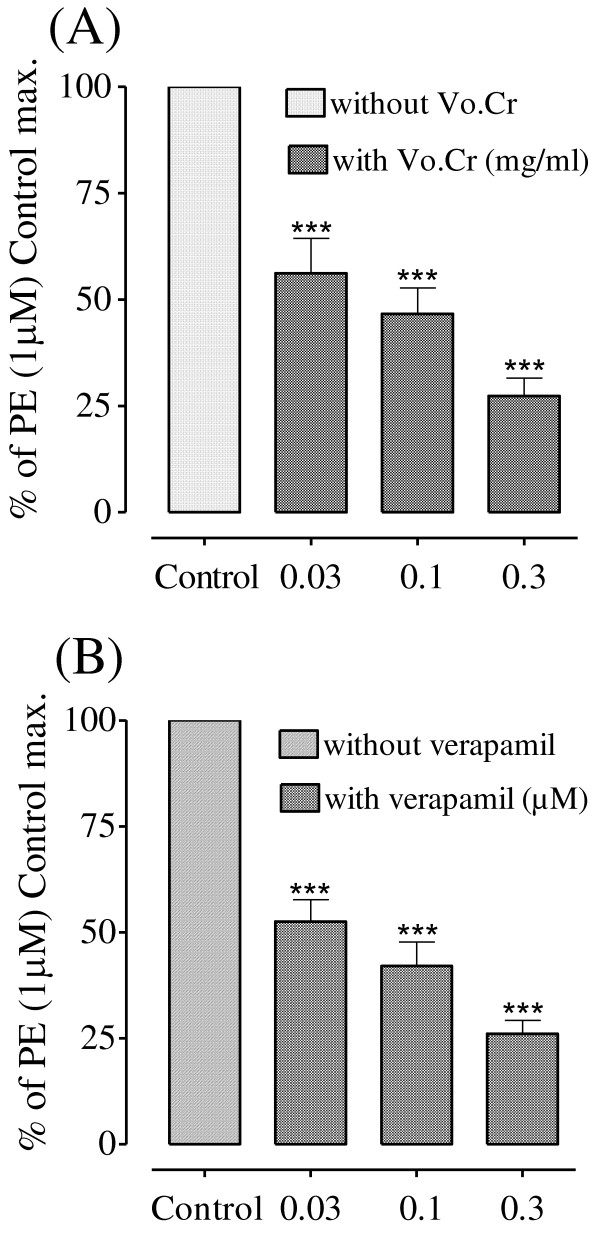
**Bar charts showing the inhibitory effects of (A) the crude extract of *Viola odorata *(Vo.Cr) and (B) verapamil on phenlyephrine responses in Ca^++ ^free Kreb's solution in isolated rat aorta preparations**. Values shown are mean ± SEM; n = 4; *** *P *< 0.001; one way ANOVA (Tukey's multiple comparison).

Vo.Cr **(**0.03-0.3) mg/ml and verapamil (0.03-0.3) μM suppressed PE (1 μM) peak responses in Ca^++ ^free Kreb's solution in a concentration-dependent manner as shown in Figure 4.

### Antidyslipidemic activities

#### Effects on tyloxapol-induced dyslipidemia

Treatment of animals with Tyloxapol (Triton WR-1339) caused an increase in serum total cholesterol and triglycerides compared to normal controls. Pretreatment of the rats with Vo.Cr at 300 and 600 mg/kg protected against tyloxapol-induced hypercholesterolemia and hypertriglyceridemia similar to atorvastatin (10 mg/kg) as shown in Table 1.

**Table 1:** Effect of *Viola odorata *(Vo.Cr) on serum total cholesterol and triglycerides in rats treated with tyloxapol.

Groups	Total cholesterol (mg/100 ml)	Triglycerides (mg/100 ml)
Control saline 10 ml/kg; i.p. (n = 6)	58.26 ± 2.83	55.21 ± 4.72

Tyloxapol 500 mg/kg; i.p. (n = 6)	621.3 ± 24.82^$$$^	5134 ± 451.8^$$$^

Tyloxapol 500 mg/kg; i.p. + Atorvastatin 10 mg/kg (n = 6)	368.2 ± 58.85***	3100 ± 297.0**

Tyloxapol 500 mg/kg; i.p. + Vo.Cr 150 mg/kg (n = 6)	495.3 ± 18.82	3841 ± 599.4

Tyloxapol 500 mg/kg; i.p.+ Vo.Cr 300 mg/kg (n = 6)	405.1 ± 50.33**	3068 ± 218.8**

Tyloxapol 500 mg/kg; i.p.+ Vo.Cr 600 mg/kg (n = 6)	351.9 ± 23.21***	2985 ± 232.1**

Values shown are mean ± SEM, n = 6

^$$$ ^*P *< 0.001; compared with normal controls.

** *P *< 0.01; *** *P *< 0.001 compared with tyloxapol controls.

(One way ANOVA followed by Tukey's post-test)

(ANOVA: analysis of variance; i.p. intraperitoneal)

#### Effects on high fat diet-induced dyslipidemia

Atherogenic diet significantly increased the serum total cholesterol (TC), low density lipoprotein-cholesterol (LDL-C) and atherogenic index, while it decreased high density lipoprotein-cholesterol (HDL-C) as compared to control. Supplementation of atherogenic diet with Vo.Cr (600 mg/kg) and atorvastatin (10 mg/kg) prevented the rise of mean serum TC, LDL-C and atherogenic index. Both the treatments significantly increased HDL-C compared to atherogenic control group. However, there was no effect seen on serum triglycerides and glucose levels. The decrease in body weight was accompanied by slight reduction in daily diet consumption. The data are summarized in Table 2.

**Table 2:** Effect of *Viola odorata *(Vo.Cr) on serum lipids, glucose, diet consumption and body weight in rats fed on atherogenic diet.

Parameters	Normal diet (n = 7)	Atherogenic diet (n = 8)	Atherogenic + Atorvastatin 10 mg/kg (n = 6)	Atherogenic + Vo.Cr 300 mg/kg (n = 7)	Atherogenic + Vo.Cr 600 mg/kg (n = 8)
Total cholesterol (mg/100 ml)	76.29 ± 5.67	295 ± 27.37^**$$$**^	129.7 ± 9.70***	211.7 ± 12.83*	140.3 ± 14.58***

HDL-C (mg/100 ml)	29.94 ± 2.70	18.10 ± 0.89^$$$^	27.06 ± 1.83*	15.35 ± 1.96	26.07 ± 1.71*

LDL-C (mg/100 ml)	31.91 ± 5.71	259.5 ± 27.4^**$$$**^	87.41 ± 8.88***	180.5 ± 12.43*	99.50 ± 14.25***

Triglycerides (mg/100 ml)	72.25 ± 4.41	86.36 ± 3.69	76.31 ± 5.82	78.99 ± 4.17	73.71 ± 2.16

Atherogenic index	1.68 ± 0.31	15.71 ± 2.01^**$$$**^	2.19 ± 0.76***	13.92 ± 1.65	4.49 ± 0.55***

Glucose (mg/100 ml)	107.4 ± 5.74	86.77 ± 6.42	113.8 ± 3.68	109.7 ± 5.68	106.8 ± 5.67

Diet consumption g/day/kg	129.4 ± 6.28	115.9 ± 3.74	101.2 ± 4.35	100.3 ± 3.15	97.58 ± 2.56*

% change in body weight	29.13 ± 2.19	44.05 ± 1.90^$$$^	34.74 ± 2.09*	34.97 ± 1.54*	33.49 ± 1.60**

Abbreviations: ANOVA: analysis of variance; HDL-C, high density lipoprotein cholesterol; LDL-C, low density lipoprotein cholesterol.

Values shown are mean ± SEM, n = 6-8

^$$$ ^*P *< 0.001 compared with normal controls

* *P *< 0.05; ** *P *< 0.01; *** *P *< 0.001 compared with atherogenic controls.

(One way ANOVA followed by Tukey's post-test)

#### Acute toxicity assessment

For acute toxicity study, mice were divided into four groups. First group received normal saline (10 ml/kg) while the other three groups were administered graded doses of 1, 3 and 5 g/kg of Vo.Cr, respectively. All animals showed usual activity in home cages and no mortality or gross behavioural changes such as apathy or aggression were observed for 48 h.

## Discussion

The aqueous-methanolic extract of *Viola odorata *caused a dose-dependent fall in blood pressure of rats under anaesthesia, which is in accordance with its medicinal use in hypertension [7]. Blood pressure is the product of cardiac output and peripheral resistance [26], hence, increase in either or both can lead to the development of hypertension. In order to explore the underlying mechanism of action, the plant extract was studied in isolated guinea-pig atria and rat aortic preparations. In guinea-pig right atria, the crude extract showed negative inotropic and chronotropic effects, similar to that caused by verapamil, a standard Ca^++ ^antagonist [27], which is known to cause cardiac depression through the inhibition of the slow inward current during the action potential plateau [28]. This indicates that the observed cardiac inhibitory effect of the plant extract might be causing a decrease in cardiac output and ultimately a fall in the blood pressure.

To characterize the vasodilator effect, when tested in pre-contracted rat aortic preparations, the plant extract inhibited both high K^+ ^(80 mM) and PE (1 μM)-induced vasoconstriction. Influx of extracellular Ca^++ ^through voltage-dependent channels (VDCs) and receptor-operated channels (ROCs) is caused by high K^+ ^and PE, respectively, resulting in increased intracellular Ca^++ ^which causes contraction [20]. This shows that the plant extract has the ability to block Ca^++ ^influx through both VDCs and ROCs. The Ca^++ ^channel blocking (CCB) activity of the crude extract was further confirmed when it shifted the Ca^++ ^concentration-response curves (CRCs) to the right with the suppression of maximum response. Verapamil, a standard CCB used clinically [29] also caused a rightward shift of the Ca^++ ^CRCs in a dose-dependent manner.

In addition to VDCs and ROCs, there is another mechanism of contraction in which Ca^++ ^influx into the cell is guided through the Ca^++ ^release from the internal stores of inositol-1, 4, 5-trisphosphate (IP_3_)-sensitive sarcoplasmic reticulum [30]. When the control responses of PE were taken in Ca^++ ^free medium, the crude extract in increasing concentrations inhibited the PE-induced peaks, indicating that it is also acting through inhibition of the intracellular Ca^++ ^channels. The results were similar to that of verapamil, suggesting the presence of CCB-like activity in the plant extract which might be responsible for its cardiac inhibitory effect in atrial preparation and the blood pressure lowering effect in anaesthetized rats though additional mechanism cannot be ruled out.

The vasodilator effect of the crude extract was further studied in isolated rat aortic preparations for its effect on vascular preparations with intact endothelium. The results showed that vasodilatation caused by Vo.Cr was reduced when the endothelium was removed, indicating some role of endothelium-dependent vasodilator mechanisms. There is a strong evidence that endothelium-derived relaxing factor (EDRF) [31] is nitric oxide (NO) synthesized in the endothelium by nitric oxide synthase (NOS) from L-arginine [32]. In order to further study the endothelium-dependent component of the crude extract, when the aortic rings with intact endothelium were pretreated with L-NAME, an inhibitor of NOS [33], the vasodilator effect was partially inhibited. The degree of relaxation induced by Vo.Cr was almost superimposable on that observed in endothelium-denuded preparations, indicating that the endothelium-dependent relaxation was through NO-dependent pathways. When the relaxant effect of the plant extract was further studied in the presence of atropine, pyrilamine and methysergide, it was found insensitive showing that the vasodilator effect of the plant extract did not involve muscarinic, histaminergic [34] or serotonergic receptors [35] respectively, which are known to have a role in receptor-mediated NO release from the endothelium (data not shown). Thus, these results indicate the involvement of other possible NO-dependent pathway(s) like the direct release of NO from the endothelial cells [36].

When the extract was tested on resting tension of endothelium-intact and denuded rat aortic rings, it caused a concentration-dependent vasoconstrictor effect. The maximum contractile effect was achieved at the concentration of 3 mg/ml which was significantly greater in the denuded tissues as compared to that in the intact ones. This again indicates the role of endothelium-derived NO which might have hindered the full expression of contractile effect as it is a potent vasodilator. When the vasoconstrictor effect of the plant extract in intact tissues was reproduced in the presence of L-NAME, the effect was augmented reaching similar to that in the denuded tissues which is in line with our findings regarding the vasodilator effect. The vasoconstrictor effect of Vo.Cr was completely blocked in intact and denuded aortic preparations when reproduced in the tissues pretreated with phentolamine, a standard α-adrenergic antagonist (data not shown). Despite the observed vasoconstrictor effect in the vascular preparation, the plant extract did not show any hypertensive effect in the intact animal, which could probably be due to some of the endogenous mediators in the whole body blocking this vasoconstrictor effect. Alternatively, the combined presence of the strong vasodilator and cardio suppressant components in the plant extract is not letting the extract to express its vasoconstrictor component in terms of an increase in the blood pressure. Whatever might be the reason, the vasoconstrictor element of the plant extract does not seem to be of any clinical significance, while evaluating its antihypertensive effect. This study is also in line with the earlier reports on *Acorus calamus *[37] and *Orchis mascula *[38] possessing a combination of vasoconstrictor and vasodilator components without showing any hypertensive activity when tested in intact animals.

Interestingly, plant extract also caused antidyslipidemic effect. In order to study the possible mode of action of the lipid-lowering activity of the plant, two different models were used. Tyloxapol-induced dyslipidemia is a widely used model to explore possible mechanism of lipid lowering drugs. It causes drastic increase in serum triglycerides and cholesterol levels due to increase in hepatic cholesterol synthesis particularly by the increase in HMG Co-A reductase (3-hydroxy-3-methyl-glutaryl Co-A reductase) activity [39] and by the inhibition of lipoprotein lipase responsible for hydrolysis of plasma lipids [40]. The plant extract caused a significant inhibition in the rise of serum triglycerides and cholesterol level, which indicates that inhibition of lipid biosynthesis, might be the possible mechanism of its lipid-lowering action.

The atherogenic or high-fat diet-induced dyslipidemia model induces a marked increase in serum total cholesterol (TC), low density lipoprotein-cholesterol (LDL-C) and atherogenic index by enhancing intestinal absorption and secretion, and decreasing catabolism of cholesterol [41]. It caused a decrease in high density lipoprotein-cholesterol (HDL-C) but did not affect serum triglyceride (TG). Treatment of the rats receiving atherogenic diet with the extract caused a significant decrease in TC and LDL-C while increased HDL-C without effecting TG and glucose levels. This may be attributed to the presence of phytochemical constituents like flavanoids and saponins in the plant. Flavanoids are reported to lower LDL-C and increase HDL-C concentrations in hypercholesteremic animals [42]. Saponins have shown to inhibit pancreatic lipase activity in high fat diet fed mice leading to greater fat excretion due to reduced intestinal absorption of dietary fats [43]. The plant extract also markedly reduced atherogenic index which is considered a better indicator of coronary heart disease risk than individual lipoprotein concentration [44]. The lipid lowering potential of the plant extract was comparable with that of atorvastatin which was used as a positive control in this study and is well known lipid-lowering drug acting via inhibition of HMG Co-A reductase [45].

Atherogenic diet also causes oxidative stress (enzymatic and non-enzymatic) in rats. It therefore increases oxidation of LDL-C which plays a key role in genesis of atherosclerosis. Antioxidants are known to effectively prevent this kind of cellular damage [46]. The presence of strong antioxidant activity in the extract [10] may offer additional benefit against oxidative stress caused by high cholesterol.

There was a significant increase in the body weights of rats on atherogenic diet compared to control group. The gain in the body weight leading to obesity is an obvious effect of such high fat diets intake [47]. It is worth mentioning that in addition to the beneficial effects of Vo.Cr in hypertension and dyslipidemia, it significantly reduced the body weight, via reducing diet intake, bringing close to the body weights of normal diet-fed rats, thus showing the weight-reducing potential of the plant extract. Obesity is amongst major health issues predisposing people to metabolic diseases such as hypertension and diabetes. According to WHO, by 2015, 2.3 billion human adults would be overweight while 700 million are expected to be obese [48]. There is poor compliance with conventional weight-management programs of increasing energy expenditure via physical activity while the drug treatment is often associated with rebound weight gain after the cessation of respective drug therapy [49]. In recent times, evaluation of therapeutic options from natural sources for treating obesity (one of the factors involved in the development of metabolic syndrome), are the focus of interest [50,51]. In this study, apparently, there was no effect on normal blood glucose level which does not rule out its potential as an anti-diabetic agent, and further studies on diabetic models are required. The antihypertensive, cardio-suppressor, vasodilator, antidyslipidemic and weight reducing properties of Vo.Cr strongly attest its usefulness in metabolic syndrome particularly if it was shown to possess anti-diabetic activity. Natural products of similar pharmacological profile are widely used in the management of metabolic syndrome [52,53].

## Conclusions

In summary, the results of this study show that the crude extract of leaves of *Viola odorata *exhibited blood pressure-lowering effect in rats under anaesthesia. In the isolated tissue preparations, the extract showed vasorelaxation mediated through inhibition of Ca^++ ^influx via membranous Ca^++ ^channels, its release from intracellular stores and NO-mediated pathways, which possibly explain the fall in BP. The plant also showed reduction in body weight and antidyslipidemic effect which may be due to the inhibition of synthesis and absorption of lipids and antioxidant activities. Thus, this study provides a pharmacologic rationale to the medicinal use of *Viola odorata *in hypertension and dyslipidemia and may be a good candidate to be developed as antihypertensive and antidyslipidemic medicine, with therapeutic potential in obesity and metabolic syndrome.

## Abbreviations

(VO.CR): *Viola odorata *leaves extract; (L-NAME): Nω-nitro-L-arginine methyl ester hydrochloride; (NO): Nitric oxide; (SD): Sprague-Dawley; (MAP) Mean arterial pressure; (DP) Diastolic pressure; (SP): Systolic pressure; (PP): Pulse pressure; (EC_50_): Effective concentration; (TC): total cholesterol; (TG): triglyceride; (LDL-C); low density lipoprotein-cholesterol; (HDL-C): high density lipoprotein-cholesterol; (VDCs): Voltage-dependent channels; (ROCs): Receptor-operated channels; (Ca^++^): calcium; (CRCs): concentration-response curves.

## Competing interests

The authors declare that they have no competing interests.

## Authors' contributions

HSS conceived and designed the study, carried out literature search and all experimental work, performed statistical analysis and data interpretation and wrote the draft of the manuscript. AHG supervised the work, raised funds and contributed intellectual input in the discussion and overall presentation of the manuscript. MHM contributed to conception and design, analysis and interpretation of data and critical review of the manuscript. NR participated in study design, helped to carry out the experiments and reviewed the manuscript. All authors have read and approved the final manuscript.

## References

[B1] Watkins LO Epidemiology and burden of cardiovascular disease Clin Cardiol 2004 27 2 6 10.1002/clc.4960271503 PMC6654722 15239484

[B2] Banos G Carvajal K Cardoso G Zamora J Franco M Vascular Reactivity and Effect of Serum in a Rat Model of Hypertriglyceridemia and Hypertension Am J Hypertens 1997 10 379 388 10.1016/s0895-7061(96)00400-1 9128203

[B3] Deshmukh M Lee HW McFarlane SI Whaley-Connell A Antihypertensive medications and their effects on lipid metabolism Curr Diab Rep 2008 3 214 220 10.1007/s11892-008-0037-7 18625119

[B4] Usmanghani K Saeed A Alam MT *Viola odorata *Linn Indusyunic Medicine 1997 Karachi: University Press 440 441

[B5] Baquar SR *Viola odorata *L Medicinal and Poisonous Plants of Pakistan 1989 1 Karachi: PRINTAS 470

[B6] Kapoor LD *Viola odorata *L Handbook of Ayurvedic Medicinal Plants 1990 Boca Raton: CRC Press 335

[B7] Keville K Rosart S *Viola odorata *L Illustrated Herb Encyclopedia 1991 New York: Michael Friedman publishing group, Inc. 207

[B8] Duke JA Bogenschutz-Godwin MJ Ducelliar J Duke PAK Sweet Violet (*Viola odorata *L.) Handbook of Medicinal Herbs. 2nd edition 2002 Boca Raton: CRC Press 715

[B9] Stuart M Stuart M Reference section The Encyclopedia of Herbs and Herbalism 1989 Spain: Macdonald & Co (Publishers) Ltd 281

[B10] Ebrahimzadeh MA Nabavi SM Nabavi SF Bahramian F Bekhradnia AR Antioxidant and free radical scavenging activity of *H. officinalis L. var. angustifolius, V. odorata, B. hyrcana and C. speciosum* Pak J Pharm Sci 2010 23 1 29 34 20067863

[B11] Vishal A Parveen K Pooja S Kannappan N Kumar S Diuretic, laxative and toxicity Studies of *Viola odorata *aerial parts Pharmacology online 2009 1 739 748

[B12] National Research Council Guide for the Care and Use of Laboratory Animals 1996 Washington: National Academy Press

[B13] Harkness JE Wagner JE The Biology and Medicine of Rabbits and Rodents, 4th edition 1995 Hagerstown: Williams and Wilkins

[B14] Ichihashi T Izawa M Miyata K Mizui T Hirano K Takagishi Y Mechanism of hypocholesterolemic action of S-8921 in rats: S-8921 inhibits ileal bile acid absorption J Pharmacol Exp Ther 1998 284 1 43 50 9435159

[B15] Evans WC Phytochemistry Trease and Evans Pharmacognosy. 5th edition 2006 Delhi: Elsevier 135 150

[B16] Ghayur MN Gilani AH Ginger lowers blood pressure through blockade of voltage-dependent calcium channels J Cardiovasc Pharmacol 2005 45 74 80 10.1097/00005344-200501000-00013 15613983

[B17] Gilani AH Shaheen F Christopoulos A Mitchelson F Interaction of ebeinone, an alkaloid from Fritillaria imperialis, at two muscarinic acetylcholine receptor subtypes Life Sci 1997 60 8 535 544 10.1016/s0024-3205(96)00691-1 9042388

[B18] Furchgott RF Zawadski JV The obligatory role of endothelial cells in the relaxation of arterial smooth muscle by acetylcholine Nature 1980 299 373 376 10.1038/288373a0 6253831

[B19] Vanhoutte PM Rubanyi GM Miller VM Houston DS Modulation of vascular smooth muscle contraction by endothelium Annu Rev Physiol 48 307 330 10.1146/annurev.ph.48.030186.001515 2871807

[B20] Karaki H Ozaki H Hori M Mitsui-Saito M Amano K Harada K Miyamoto S Nakazawa H Won KJ Sato K Calcium movements, distribution, and functions in smooth muscle Pharmacol Rev 1997 49 157 230 9228665

[B21] Hashimoto M Hirata M Itoh T Kanmura Y Kuriyama H Inositol 1, 4, 5-triphosphate activates pharmaco-mechanical coupling in smooth muscle of rabbit mesenteric artery J Physiol 1986 370 605 618 10.1113/jphysiol.1986.sp015953 PMC1192699 3007748

[B22] Khanna AK Rizvi F Chander R Lipid lowering activity of *Phyllanthus niruri *in hyperlipemic rats J Ethnopharmacol 2002 82 1 19 22 10.1016/s0378-8741(02)00136-8 12169400

[B23] Berroughui H Ettaib A Herrera Gonzalez MD Alvarez de Sotomayor M Bennari-Kabchi N Hmamouchi M Hypolipidemic and hypocholesterolemic effect of argan oil (*Argan spinosa *L.) in Meriones Shawi rats J Ethnopharmacol 2003 89 1 15 18 10.1016/s0378-8741(03)00176-4 14522427

[B24] Mandukhail SU Aziz N Gilani AH Studies on antidyslipidemic effects of *Morinda citrifolia *(Noni) fruit, leaves and root extracts Lipids Health Dis 2010 9 88 10.1186/1476-511X-9-88 PMC2939587 20727145

[B25] Gilani AH Khan AU Ghayur MN Ali SF Herzig JW Antispasmodic effects of Rooibos tea (*Aspalathus linearis*) is mediated predominantly through K^+ ^-channel activation Basic Clin Pharmacol Toxicol 2006 99 365 373 10.1111/j.1742-7843.2006.pto_507.x 17076689

[B26] Johansen PL Hemodynamic effects of calcium antagonists in hypertension Calcium Antagonists in Clinical Medicine 1992 Philadelphia 62 98

[B27] Fleckenstein A Specific pharmacology of Ca^++ ^in myocardium, cardiac pacemakers and vascular smooth muscle Annu rev of pharmacol 1977 17 149 166 10.1146/annurev.pa.17.040177.001053 326161

[B28] Roden DM Antiarrhythmic drugs Goodman and Gilman's the Pharmacological Basis of Therapeutics 2006 11 New York: McGraw-Hill 899 932

[B29] Godfraind T Miller R Wibo M Calcium antagonism and calcium entry blockade Pharmacol Rev 1986 38 312 416 2432624

[B30] Benham CD Bolton TB Lang RJ Takewaki T Calcium-activated potassium channels in single smooth muscle cells of rabbit jejunum and guinea-pig mesenteric artery J Physiol 1986 371 45 67 10.1113/jphysiol.1986.sp015961 PMC1192710 2422353

[B31] Furchgott RF Zawadski JV The obligatory role of endothelial cells in the relaxation of arterial smooth muscle by acetylcholine Nature 1980 299 373 376 10.1038/288373a0 6253831

[B32] Palmer RM Ashton DS Moncada S Vascular endothelial cells synthesize nitric oxide from L-arginine Nature 1988 333 664 666 10.1038/333664a0 3131684

[B33] Fantel AG Nekahi N Shepard TH Cornel LM Unis AS Lemire RJ The teratogenicity of N^G ^-nitro-L-arginine methyl ester (L-NAME), a nitric oxide synthase inhibitor in rats Reprod Toxicol 1997 11 709 717 10.1016/s0890-6238(97)00033-6 9311580

[B34] Chen GF Suzuki H Direct and indirect actions of acetylcholine and histamine on intrapulmonary artery and vein muscles of the rat Jpn J Physiol 1989 39 1 51 65 10.2170/jjphysiol.39.51 2724668

[B35] Saxena PR Villalón CM Cardiovascular effects of serotonin agonists and antagonists J Cardiovasc Pharmacol 1990 15 Suppl 7 S17 34 1702484

[B36] Tanner MA Bu X Steimle JA Myers PR The direct release of nitric oxide by gypenosides derived from the herb *Gynostemma pentaphyllum* Nitric Oxide 1999 3 5 359 365 10.1006/niox.1999.0245 10534439

[B37] Shah AJ Gilani AH Blood pressure-lowering and vascular modulator effects of *Acorus calamus *extract are mediated through multiple pathways J Cardiovasc Pharmacol 2009 54 1 38 46 10.1097/FJC.0b013e3181aa5781 19528816

[B38] Aziz N Mehmood MH Siddiqi HS Mandukhail SU Sadiq F Maan W Gilani AH Antihypertensive, antidyslipidemic and endothelial modulating activities of *Orchis mascula* Hypertens Res 2009 32 11 997 1003 10.1038/hr.2009.148 19745827

[B39] Kuroda M Tanzawa K Tsujita Y Endo A Mechanism for elevation of hepatic cholesterol synthesis and serum cholesterol levels in Triton WR-1339-induced hyperlipidemia Biochimica Biophysica Acta 1977 489 119 125 10.1016/0005-2760(77)90238-7 911870

[B40] Schotz MC Scanu A Page IH Effect of Triton on lipoprotein lipase of rat plasma Am J Physiol 1957 188 399 402 10.1152/ajplegacy.1957.188.2.399 13411223

[B41] Heuman DM Vlahcevic ZR Bailey ML Hylemon PB Regulation of bile acid synthesis. II. Effect of bile acid feeding on enzymes regulating hepatic cholesterol and bile acid synthesis in the rat Hepatology 1988 8 892 897 10.1002/hep.1840080431 3391517

[B42] Daniel RS Devi KS Augusti KT Sudhakaran Nair CR Mechanism of action of antiatherogenic and related effects of *Ficus bengalensis *Linn. flavonoids in experimental animals Indian J Exp Biol 2003 41 4 296 303 15255637

[B43] Han LK Zheng YN Xu BJ Okuda H Kimura Y Saponins from *platycodi radix *ameliorate high fat diet-induced obesity in mice J Nutr 2002 132 8 2241 2245 10.1093/jn/132.8.2241 12163669

[B44] Kinosian B Glick H Press L Purer KL Cholesterol and coronary heart disease: predicting risks in men by changes in levels and ratios J Invest Med 1995 43 443 450 8528755

[B45] Furman A Meier JL Malmstrom RA Lopez JR Schaefer S Comparative efficacy of ezetimibe/simvastatin, rosuvastatin, and atorvastatin in uncontrolled hyperlipidemia patients Am J Manag Care 2011 17 8 538 44 21851141

[B46] Vijayakumar RS Surya D Nalini N Antioxidant efficacy of black pepper (*Piper nigrum *L.) and piperine in rats with high fat diet induced oxidative stress Redox Rep 2004 9 105 110 10.1179/135100004225004742 15231065

[B47] Thounaojam M Jadeja R Ansarullah Devkar R Ramachandran AV Dysregulation of lipid and cholesterol metabolism in high fat diet fed hyperlipidemic Rats: Protective Effect of *Sida rhomboidea*. roxb leaf extract J Health Sci 2009 55 413 420

[B48] World Health Organization Obesity and overweight 2006 http://www.who.int/mediacentre/factsheets/fs311/en/index.html Ref Type: Online Source

[B49] Hasani-Ranjbar S Nayebi N Larijani B Abdollahi M A systematic review of the efficacy and safety of herbal medicines used in the treatment of obesity World J Gastroenterol 2009 15 25 3073 3085 10.3748/wjg.15.3073 PMC2705729 19575486

[B50] Alarcon-Aguilar FJ Zamilpa A Perez-Garcia MD Almanza Perez JC Romero-Nuñez E Campos-Sepulveda EA Vazquez-Carrillo LI Roman-Ramos R Effect of *Hibiscus sabdariffa *on obesity in MSG mice J Ethnopharmacol 2007 114 66 71 10.1016/j.jep.2007.07.020 17765418

[B51] Jeon WK Kim JH Lee HW Ko BS Kim HK Effects of *Rhus verniciflua *Stokes (RVS) extract on diet-induced obesity in C57BL/6 mouse Kor J Pharmacognosy 2003 34 339 343

[B52] Shahraki MR Harati M Shahraki AR Prevention of high fructose-induced metabolic syndrome in male wistar rats by aqueous extract of *Tamarindus indica *seed Acta Med Iran 2011 49 5 277 283 21713743

[B53] Shih CC Lin CH Lin WL Effects of *Momordica charantia *on insulin resistance and visceral obesity in mice on high-fat diet Diabetes Res Clin Pract 2008 81 134 143 10.1016/j.diabres.2008.04.023 18550200

